# Episodic and semantic feeling-of-knowing in aging: a systematic review and meta-analysis

**DOI:** 10.1038/s41598-023-36251-9

**Published:** 2023-09-30

**Authors:** Méline Devaluez, Audrey Mazancieux, Céline Souchay

**Affiliations:** 1 LPNC, CNRS, UMR 5105, Université Grenoble Alpes, Grenoble, France; 2 Cognitive Neuroimaging Unit, NeuroSpin Center, Institute for Life Sciences Frédéric Joliot, Fundamental Research Division, Commissariat à l’Energie Atomique et aux Énergies Alternatives, INSERM, Université Paris-Sud, Université, Paris-Saclay, Gif-sur-Yvette, France

**Keywords:** Human behaviour, Consciousness

## Abstract

A complex pattern of preservation and deterioration in metacognition in aging is found, especially regarding predicting future memory retrieval (i.e., feeling-of-knowing, FOK). While semantic FOK (sFOK) is preserved with age, studies on episodic tasks (eFOK) produce equivocal findings. We present a meta-analysis of 20 studies on eFOK and sFOK, analyzing the difference in metacognitive sensitivity between 922 younger and 966 older adults, taking into account the difference in memory performance. The sFOK studies yielded no overall age effect (8 effects, *g* = −0.10 [−0.29, 0.10]). However, we found a reliable age-group difference on eFOK (22 effects, *g* = 0.53 [0.28, 0.78]), which was moderated when considering recognition performance. Moreover, using aggregated data of 134 young and 235 older adults from published and unpublished studies from our lab, we investigated memory performance as an explanation of the eFOK deficit. We show that older adults are less metacognitively sensitive than younger adults for eFOKs which is, at least partly, due to the age-related memory decline. We highlight two non-exclusive explanations: a recollection deficit at play in the first and second order tasks, and a confound between first order performance and the measure used to assess metacognitive sensitivity.

## Introduction

Metacognition, the ability to monitor and control our cognitive ability, is multifaceted. It has been investigated in the context of several domains (e.g., episodic memory, semantic memory, perception) or on several levels (local or global^1^). Deficient metacognition has been shown to occur in various populations from people with neurological diseases^2^ to psychiatric populations^3^. In healthy aging, the question whether metacognition is altered is controversial. Although the alteration of frontoparietal networks in aging is known to involve regions implicated in metacognition^4,5^, empirical evidence for such a deficit remains inconsistent. The modifications of metacognition abilities in healthy aging is to be distinguished from patterns of metacognitive deterioration in age-related disorders (e.g., Alzheimer’s disease^6^; Pakinson’s disease^7^). The question of whether metacognition is intact in healthy aging is of importance, as it would be the basis for older adults (OA) to use compensatory strategies for any cognitive decline.

We aim to address the status of metacognition in healthy aging by focusing on a meta-analysis for one specific task—the feeling-of-knowing (FOK). The FOK is the ability to predict future recognition of currently unrecallable information. Numerous studies have shown that OA make inappropriate evaluations of their memory for recently learned information on such a task. It is of particular interest since in most other experimental paradigms OA and younger adults (YA) show equivalent metacognition. Age invariance (or even superior performance in OA) in metacognitive sensitivity (i.e., the ability to discriminate between correct and incorrect responses) has been reported for retrospective confidence tasks^8–10^, and for Judgement-Of-Learning (JOL) and delayed-JOL paradigms^11–13^. Similarly, recent work has shown preserved metacognitive sensitivity confidence judgments in this population^14^. Thus, OA’s metacognitive sensitivity on episodic FOK (eFOK) tasks is of interest both for how we understand the cognitive aging process, but also how we conceive metacognition as being more general beyond a specific type of metacognitive judgment or beyond the memory domain in a specific type of judgment.

**Figure 1:**
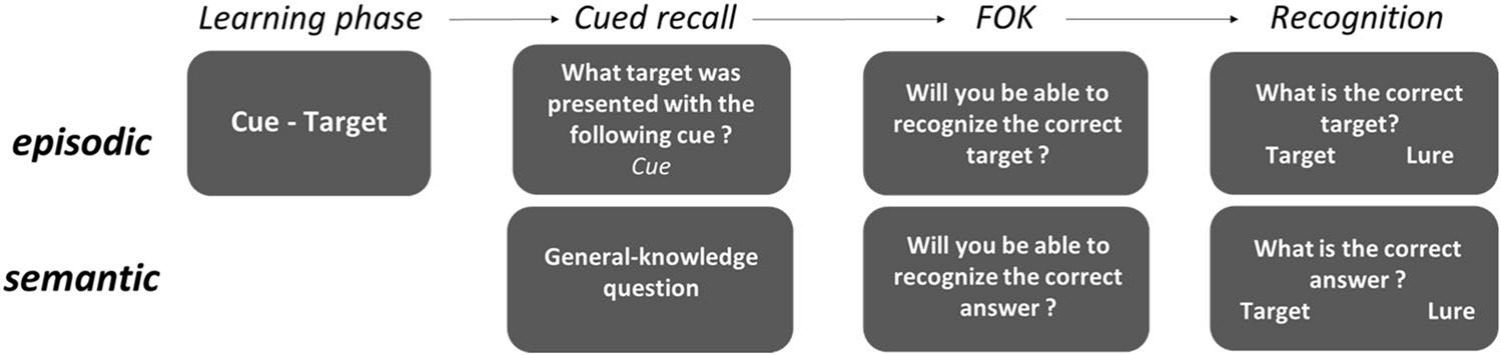
Basic episodic (top) and semantic (bottom) FOK paradigms. FOKs are made after a cued-recall attempt in both cases and are made on a “yes/no” or a Likert-scale. The recognition can be an “old/new” task or a multiple-alternative forced-choice. First-order performance is usually the proportion of correct recognition. Metacognitive sensitivity is assessed by comparing FOKs with memory performance in the recognition phase.

This idea refers to the notion of the domain-generality of metacognition. From this perspective, metacognition is not entirely encapsulated in each cognitive domain but some general abilities are shared across cognitive domains^15,16^. Within FOK judgements, a contrast is made between semantic and episodic FOK (see Fig. 1). In the eFOK, there is a first study phase using cue-target pairs followed by a FOK phase where the participants recall target words when given cues and/or make a prediction of future recognition. In semantic FOK (sFOK) tasks, there is no study phase but only an immediate FOK phase where the participant attempts to recall answers to general knowledge questions. In both tasks metacognitive sensitivity is assessed by comparing the FOK predictions with final recognition memory.

The accuracy of OA’s metacognitive evaluations on episodic tasks can either be contrasted with YA’s performance on the same episodic task, or by comparing episodic and semantic materials. The claim is often made that whereas there is metacognitive sensitivity age-equivalence on the sFOK task, there are age differences on eFOK tasks^17–19^. This is found across several different neuropsychological populations (e.g., in autism spectrum disorder^20^; in Alzheimer’s disease^19,21^). Similarly, in a large sample of university students it was shown that whereas the sensitivity of retrospective confidence judgements was correlated across semantic and episodic tasks, the same pattern broke down for FOKs—a challenge for the concept of domain generality of metacognition^16^. In the same way, a recent meta-analysis on neuroimaging studies showed different cerebral areas involved in prospective (e.g., JOLs and FOKs) and retrospective metacognitive judgements^5^. Comparing FOKs for semantic and episodic material, a neuroimaging study inspired by the neuropsychological approach has shown different patterns of neural activations for the two FOK judgements^22^.

A few critical theoretical issues concerning metacognition in OA and the domain generality of metacognition therefore rest on the finding that metacognitive sensitivity of eFOK (which we will refer to as eFOK sensitivity) is differentially impaired in OA. However, this is far from an unequivocal finding, with some studies suggesting age equivalence^23–26^ and some studies reporting age differences^27–29^. One challenge is to better understand the discrepancies in the literature. Overall, studies addressing the eFOK sensitivity in relation to aging used similar paradigms and populations of interest, but they also show differences in methods (e.g., type of material, study time, FOK scale) and diverse conclusions that we review in the “Results” section. Of great importance here, some authors have taken additional steps to ensure equivalent memory performance between age-groups while others did not try to control for first order performance.

This difference is particularly relevant as poorer episodic memory is one of the main hypotheses that is proposed to account for the eFOK sensitivity deficit in aging^26^. In fact, multiple theories have been proposed to explain the potential specific impairment of eFOK in aging. One theory postulates that the age-related eFOK sensitivity and episodic memory impairments are driven by an executive function deficit^30^. Support for this theory comes from results showing a correlation between eFOK sensitivity and executive function test scores^27,31^ and regression analyses suggesting executive functioning as a main factor of eFOK sensitivity^32^. These results are in line with functional neuroimaging results suggesting the importance of a fronto-temporal network and the critical involvement of the prefrontal cortex in eFOK^33^, which might underlie a monitoring process involving interactions between executive and memory functions.

A deficit in self-initiated processes has also been proposed as the basis for the decrease in eFOK sensitivity with aging^34^. These authors propose that the quality of partial information accessed about the target when making the FOK judgment is a determinant of FOK sensitivity. OA displayed better sensitivity when they had access to correct partial information. Thomas et al.^34^ suggest that the eFOK sensitivity deficit observed in aging is thus associated with less effective use of partial information. Finally, two other theories advance that memory processes underpin the eFOK decline with aging. The memory constraint hypothesis^26^ proposes that the decrease in FOK sensitivity on episodic memory tasks is a consequence of the quality of original encoding. Poorer encoding leads to weaker memory strength and does not allow enough information for accurate FOKs. Other work^19^ suggests that this eFOK deficit is a consequence of a lack of recollection (of contextual or retrieval cues) during the recall attempt made at the moment of the FOK. OA make less accurate predictions because they fail to recollect the target or any information that could lead them to believe they know the target. This result has been recently strengthened by a study showing that metacognition efficiency (i.e., metacognitive sensitivity that controls for recognition memory performance) is correlated with recall performance during the FOK phase^16^.

As memory processes contribute to the eFOK deficit, a challenge is to examine whether the observed eFOK deficit in aging is a direct consequence of the episodic memory decline associated with age. The relationship between first order (i.e., memory) and second order (i.e., metacognitive) performance is a critical point and a source of debate in the field of metacognition. Several classical measures of metacognitive sensitivity have proven to correlate with first order performance (e.g., Goodman-Kruskall gamma correlation; see^35^ for a review). As such, the question raised above becomes particularly interesting given that the literature about FOK in aging relies strongly on the gamma correlation^36,37^. Gamma correlations measure the degree of relationship between the accuracy of a response given during the first order task and the metacognitive judgment for the same trial. Therefore, a strong unbalance between correct and incorrect responses in the first order task biases the gamma correlation. Similarly, a high proportion of high or low FOK responses also strongly modifies its value. This second issue suggests more specifically that the eFOK deficit observed in aging could also be the result of a metacognitive bias different from YA’s (i.e., over- or under-confidence).

We present a systematic review of published studies conducted on eFOK and sFOK sensitivity in aging. In the first part, using a meta-analytic approach, one goal was to assess the general age-related performance on both episodic and semantic tasks in the existing literature. The issue of a confound between eFOK sensitivity and memory performance is not a new concern, and as such several published studies have taken additional steps to limit memory inequality between age groups, by either constraining YA or favoring OA’s memory performance. We explored whether this specific deterioration in eFOK sensitivity might be a consequence of episodic memory impairment as it has been done for other populations^38^.

In a second part, using an aggregated dataset from published and unpublished studies conducted in our lab, we aimed at providing additional evidence of the contribution of first order performance to metacognitive sensitivity by matching participants according to memory performance using either recall or recognition. Knowing the limits of standard measures of FOK sensitivity, we also intended to evaluate eFOK sensitivity in OA and YA using classical gamma correlations as well as measures less contaminated by metacognitive bias (e.g., type-2 d’). In both parts, we expected a reduced difference in eFOK sensitivity between OA and YA when first order performance (recall or recognition) was controlled for than when it was not considered.

## Results

### Systematic review and global meta-analysis

#### Qualitative review

**Figure 2:**
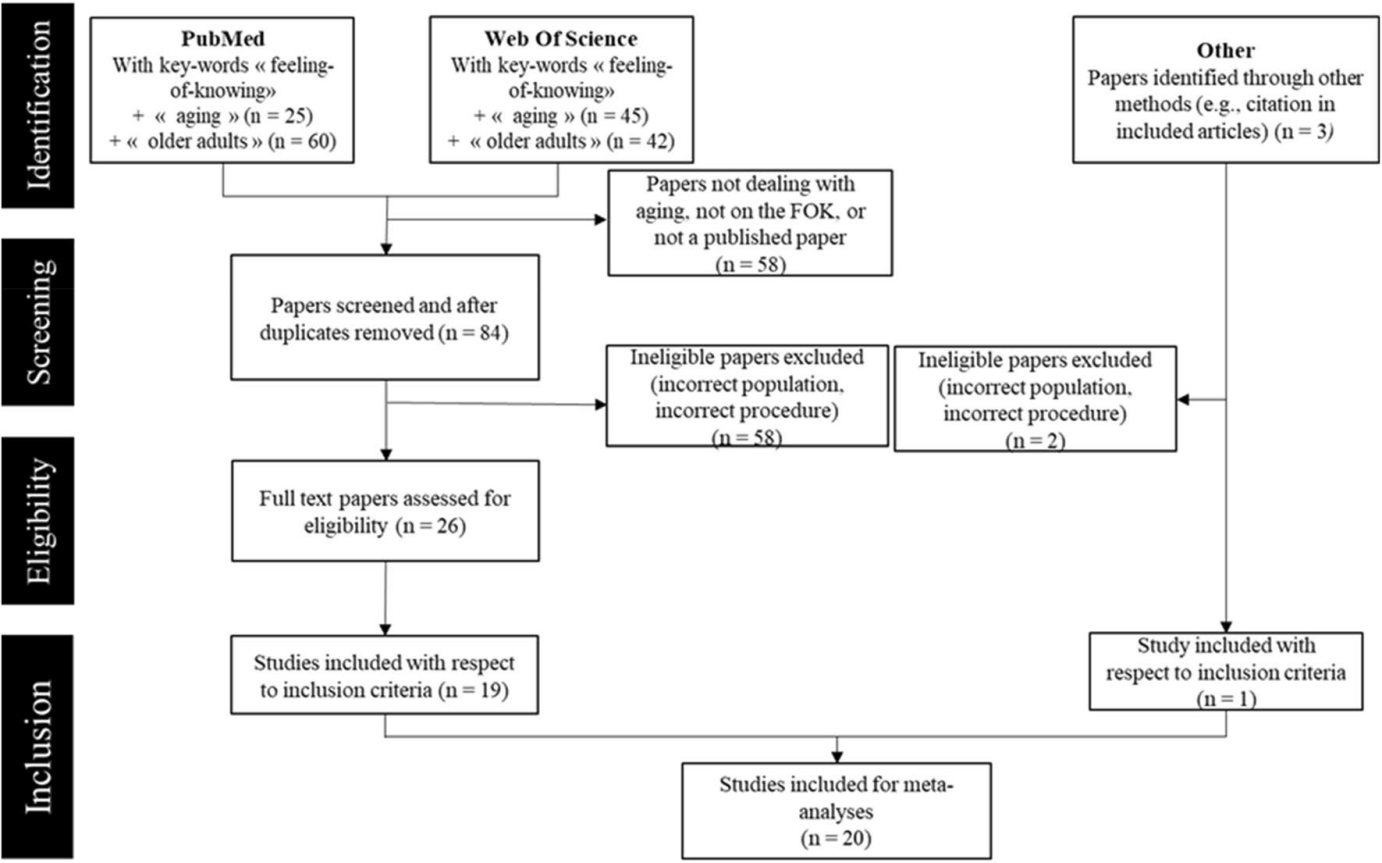
Flow chart of the steps for identification and selection of records included in the systematic review and meta-analysis.

In total, 22 effects taken from 20 experiments were included in the meta-analysis (see Fig. 2 and “Method” section for the steps of identification and selection of records). Table 1 provides a summary of the studies investigating FOK sensitivity in healthy aging which were included. Thirteen studies explored the age-related effect on the eFOK only, three on the sFOK only, and four considered both the eFOK and sFOK. All studies compared at least one OA group to one YA group. Some studies included multiple groups of one age-category because authors tried to compare groups both when memory performance was equated and when it was not.

**Table 1:** Summary of all studies included in each of the two global meta-analyses.

Authors (year)		Sample size		Age range		Task	Material	Recognition type	FOK scale	Number of trials	Attempt of matched performance
		YA	OA	YA	OA						
Allen-Burge and Storandt (2000)^42^—Exp 1		45	45	18–21	62–79	Semantic	Rare-word definitions	2AFC	7-point	50	No
Butterfield et al. (1988)^43^—Exp 2		54	36	18–19	60–93	Semantic	General-knowledge questions	7AFC	Yes/no	12	No
Douchemane et al. (2007)^17^		18	18	20–38	60–80	Semantic	Definitions of words	5AFC	Yes/no	40	No
						Episodic	Pairs of words	5AFC	Yes/no	40	No
Eakin and Hertzog (2012)^24^		51	42	NA	NA	Episodic	Pairs of words	5AFC	0–100%	44 (22 + 22)	No
Eakin et al. (2014)^23^		50	56	18–21	NA	Semantic	Famous faces’ names	3AFC	0–100%	30	No
						Episodic	Non-famous faces with names	3AFC	0–100%	30	No
Hertzog et al. (2010)^26^		54	54	NA	NA	Episodic	Pairs of words	4AFC	0–100%	20	Manipulation of the delay between encoding and test. Larger delay for the younger group (7 days). Two OA groups: one with a delay of 48 h and the other with a delay of 30 min*
MacLaverty and Hertzog (2009)^41^		206	200	17–27	60–80	Episodic	Pairs of words	4AFC	25%-100%	36	No
Marquié and Huet (2000)^9^		22	22	18–30	61–77	Semantic	General-knowledge + computer-related questions	4AFC	5-point	138 (69 + 69)	No
Morson et al. (2015)^18^		35	16	18–29	60–85	Semantic	General-knowledge questions	4AFC	Yes/no	60	No
						Episodic	Answers to general-knowledge questions (unrecognized in semantic memory)	4AFC	Yes/no	60 minus items with correct recognition on the sFOK task	No
Perrotin et al. (2006)^31^		40	62	20–30	61–89	Episodic	Pairs of words	5AFC	Yes/no	40	No
Sacher et al. (2013)^39^	Full attention	20	60	22–36	61–82	Episodic	Pairs of words	5AFC	6-point (0–100%)	60	Manipulation of attention. Three YA groups: one group with divided attention at encoding, the other with divided attention for FOK judgment. The third group was a control group with full attention
	Attention encoding	20									
	Attention FOKs	20									
Sacher et al. (2015)^29^		59	61	20–36	61–82	Episodic	Pairs of words	5AFC	0–100%	60	No
Souchay and Isingrini (2012)^28^		16	36			Episodic	Pairs of words	Old/new	Yes/no	40	No
Souchay et al. (2000)^27^		20	41	20–32	60–98	Episodic	Pairs of words	Yes/no	Yes/no	36	No
Souchay et al. (2002)^25^		16	16	21–30	52–93	Episodic	Pairs of words	Old/new	Yes/no	20	No
Souchay et al. (2007)^19^^—^Exp 1		20	40	20–30	64–91	Semantic	General-knowledge questions	5AFC	Yes/no	40	No
						Episodic	Pairs of words	5AFC	Yes/no	40	No
Souchay et al. (2007)^19^^—^Exp 2		20	36	20–30	60–91	Episodic	Pairs of words	5AFC	Yes/no	40	No
Thomas et al. (2011)^40^^—^Exp 1		42	42	18–24	61–82	Episodic	Pairs of words	6AFC	17–100%	36	Manipulation of the presentation time at encoding (500 ms for YA and 5 s for OA). Participants could only move to the FOK phase if they had at least 33% of correct recall
Thomas et al. (2011)^40^^—^Exp 2	Group info before	22	20	18–24	65–82	Episodic	Pairs of words	6AFC	17–100%	36	Same as Experiment 1
	Group info after	24	15								
Thomas et al. (2011)^40^^—^Exp 3	Group FOK deadline	24	24	18–24	66–85	Episodic	Pairs of words	6AFC	17–100%	36	Same as Experiments 1 and 2
	Group info deadline	24	24								

*OA* older adults, *YA* young adults, *XAFC* X-alternative-forced-choice task, *NA* not available.

*This 30-min condition was not included in the meta-analysis as data were missing.

In the papers selected for review, 12 studies described a significant age-related deficit in FOK sensitivity (^27,39^; 3 experiments in^18,29,40^; 2 experiments in^17,19,28,31^). Two of these reports also showed reduced or no significant age-related difference in eFOK sensitivity under conditions allowing equated memory performance between YA and OA groups^39^ or when memory performance (recall or recognition) was controlled for in the analyses^28^. On the contrary, four studies reported a significant age effect on eFOK sensitivity despite memory performance being equated between age groups (^18^; experiments 1 & 2 of^40^ for equal performance on both recall and recognition; Experiment 3 of^40^ for equal performance only on recognition).

Additionally, four studies reported no significant age effect on eFOK sensitivity^23,24,26,41^. Memory performance was equated between YA and OA in one of those studies^26^. Hertzog et al.^26^ manipulated delay between encoding and test for both age-groups allowing less delay to OA (48 h for one group and 30 min for the other) than to YA (7 days). The two groups which had matched episodic recognition performance (48-h delay OA and YA group) did not show significant difference in eFOK sensitivity. The number of times items were presented at encoding was also manipulated. Notably, for items only presented once, YA showed better eFOK sensitivity than OA, despite memory performance being matched.

No study reporting sFOK showed a significant age effect on sFOK sensitivity (^18,23^; Experiment 1 in^9,19^; Experiment 1 of^17,42^), although memory performance was sometimes also unmatched as OA performed worse than YA (e.g.,^17,19^ when education is not controlled for) or even better than YA^18,43^.

#### Global meta-analyses

**Figure 3:**
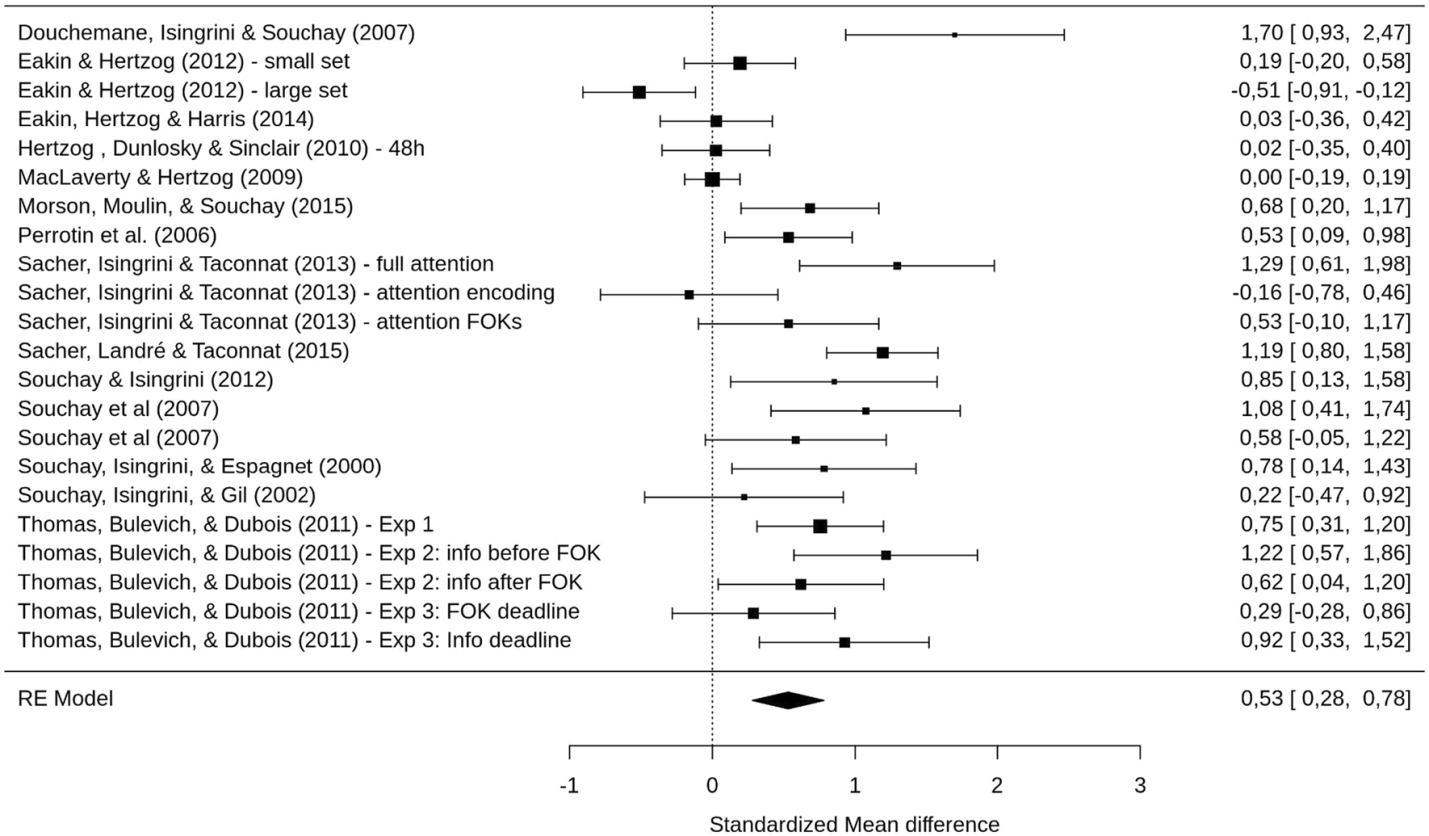
Forrest plot of the effect of eFOK deficit in OA. Confidence interval of the overall estimated effect does not overlap with 0.

We performed several hierarchical meta-analytic models (see “Method” section) on the metacognitive sensitivity measure (gamma correlations). The first model (eM1) estimated the overall effect-size of a difference in metacognitive sensitivity between YA and OA for eFOK. It showed lower gamma correlations in OA compared to YA, *g* = 0.53 [0.28, 0.78], *p* < 0.001 (see Fig. 3). The total heterogeneity analysis revealed a significant Q-statistic (*Q*(df = 21) = 102.77, *p* < 0.001). As we used a multilevel meta-analytic model, the amount of heterogeneity was computed for each level^44^ of the model (i.e., each random effect: studies, experiments, and effects). *I*^2^ for each level corresponds to 54.23%, 0%, and 24,86% respectively leading to 79.09% of the total variance due to heterogeneity.

**Figure 4:**
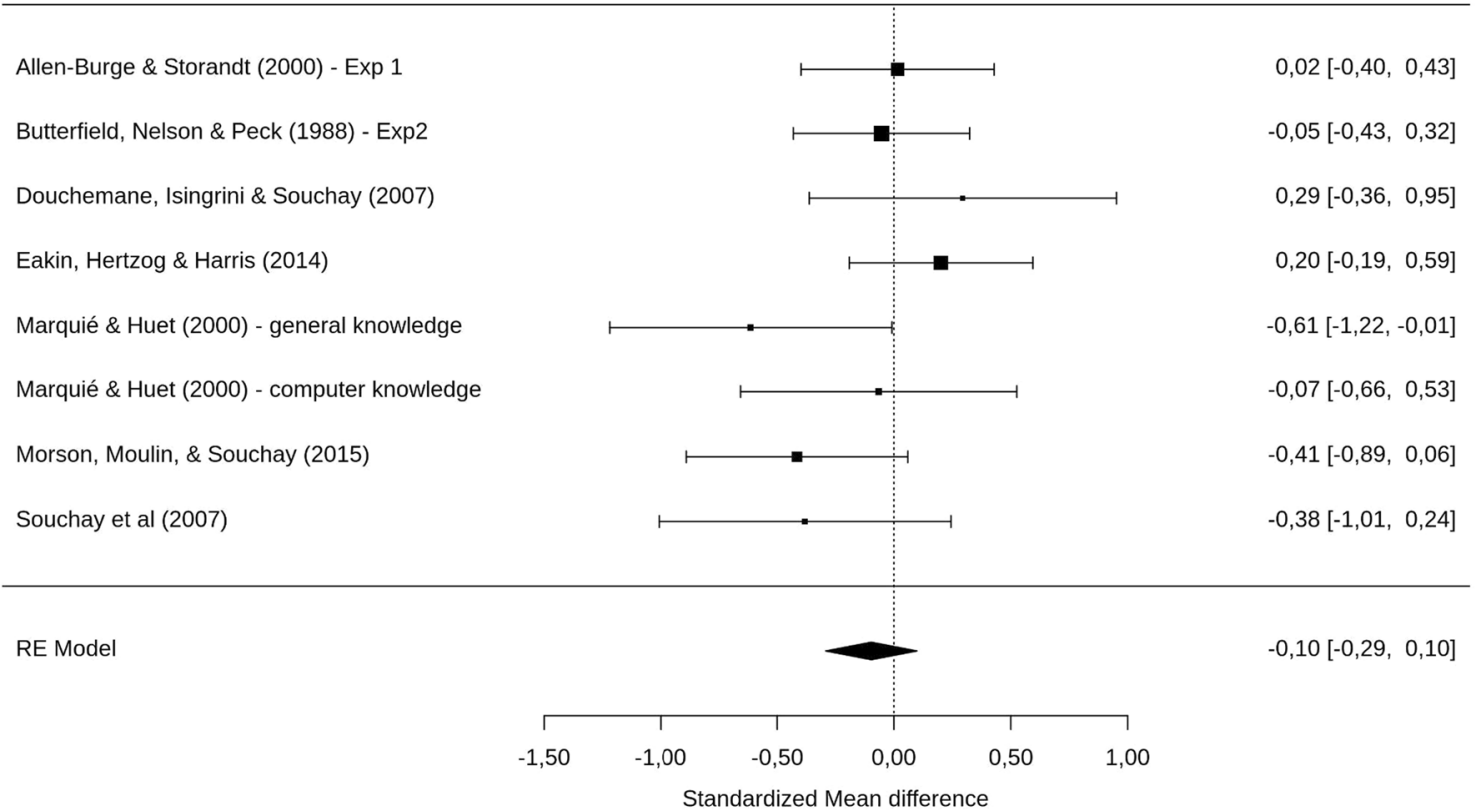
Forrest plot showing an absence of sFOK deficit in OA. Confidence interval of the overall estimated effect does overlap with 0.

The same model for sFOK (sM2) showed no age effect on gamma correlation across studies, *g* = −0.10 [−0.29, 0.10], *p* = 0.330 (see Fig. 4). The total heterogeneity analysis was non-significant, *Q*(df = 7) = 9.24, *p* = 0.236. That is, we found no age difference in gamma correlations for the sFOK tasks.

According to our preregistration and because of significant heterogeneity in our eFOK model, we tested a moderation by memory performance. The M2 model tested the moderation of recall and revealed only a trend moderation effect, *QM*(df = 1) = 3.53, *p* = 0.060. On the contrary, the M3 model tested the moderation of recognition and revealed a significant moderation effect, *QM*(df = 1) = 5.14, *p* = 0.023. Moreover, for a recognition effect size of 0 (no group difference), the estimated effect does not reach significance (estimate = −0.24, *p* = 0.514). Finally, the M4 model tested the moderation of type of recognition and showed no effect of this moderator, *QM*(df = 1) = 0.52, *p* = 0.469.

**Figure 5:**
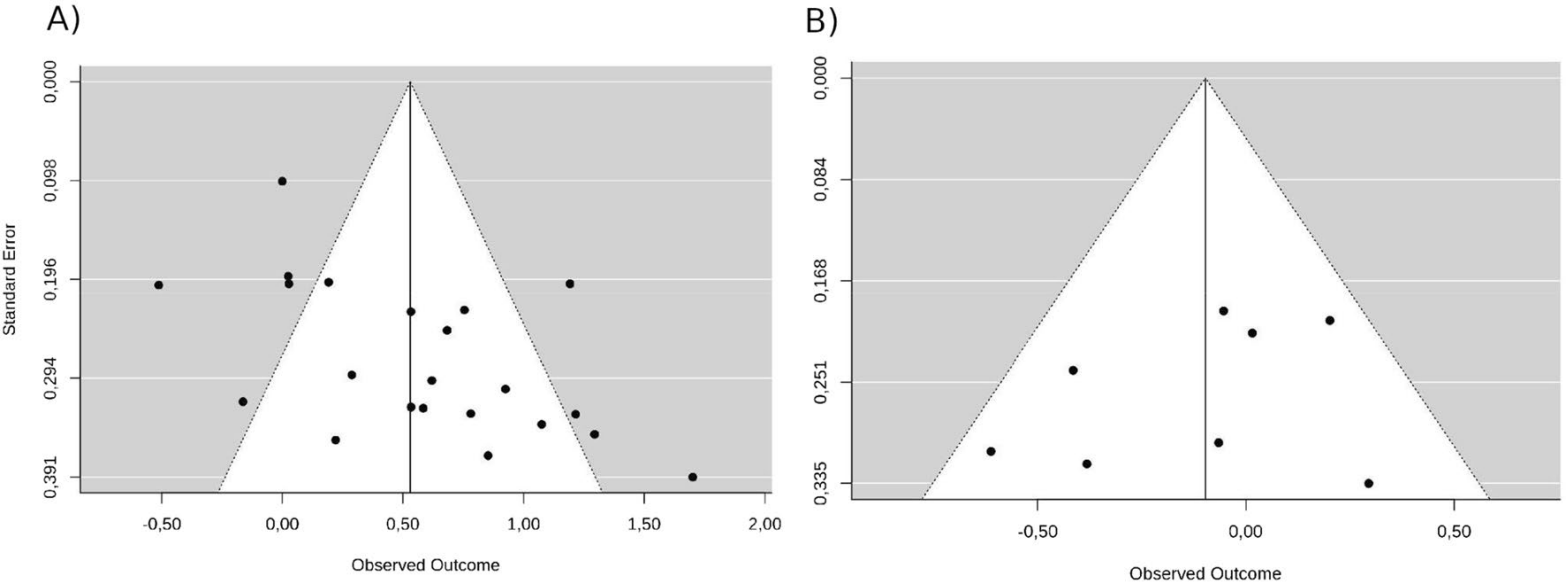
Funnel plot centered on the overall effect size (vertical line) for eFOK model (**A**) and sFOK model (**B**). The white areas are the 95% confidence intervals of the overall effect sizes. Points in the gray areas are outliers.

For the sFOK model, no publication bias was identified as the shape of the funnel plot showed no asymmetry (*z* = −0.91, *p* = 0.365). However, for the eFOK model, a significant asymmetry in the funnel plot was identified for eFOK: *z* = 2.99, *p* = 0.003) suggesting a publication bias (see Fig. 5).

### Analyses of individual scores from aggregated dataset

#### Qualitative review

Data was taken from five published (^28,31^; two studies from^19,27^ and 1 unpublished studies conducted in our lab (note that the 5 published studies were also included in the global meta-analysis). In total, data from 235 OA (*M*_*age*_ = 72.49; *SD*_*age*_ = 8.90) and 134 YA (*M*_*age*_ = 24.91, *SD*_*age*_ = 3.14) were analyzed. All studies used a standard eFOK paradigm split into 3 parts. Participants first studied pairs of cue-target words for 5 s. Following the learning phase, participants were presented with each cue and were given up to 15 s to recall the target associated with it. After each recall attempt, they made an FOK judgment. They were asked to say “yes” when they thought they would be able to recognize the target later and “no” when they thought they would not be able to recognize it. Finally, participants were asked to identify the target among distractors. Four studies used a five-alternative forced-choice paradigm in which each target was presented with four distractors and participants were asked to select the target. The two other studies presented a list of all targets together with the same number of distractors in which participants were asked to identify all targets. The six experiments also slightly differed in the number of trials (36 or 40 word pairs).

#### Meta-analyses

**Figure 6:**
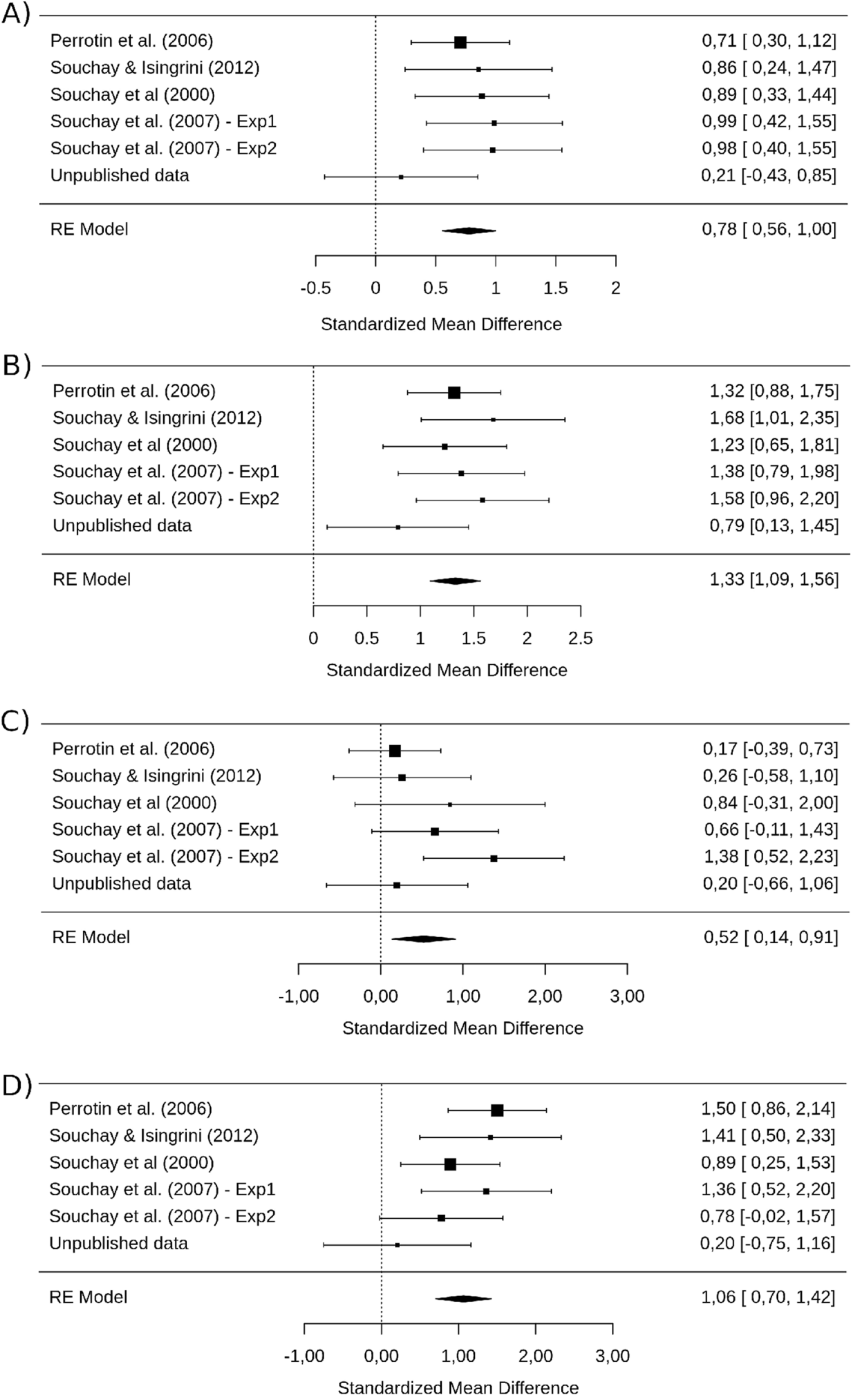
**(A)** Forrest plot of the effect of eFOK deficit in OA. Confidence interval of the overall estimated effect does not overlap with 0. (**B**) Forrest plot of the effect of recall deficit in OA. Confidence interval of the overall estimated effect does not overlap with 0. **(C)** Forrest plot of the effect of eFOK deficit in OA for half of OA with the best recall performance and half of YA with the worst recall performance. Confidence interval of the overall estimated effect does not overlap with 0. **(D)** Forrest plot of the effect of eFOK deficit in OA for half of OA with the worst recall performance and half of YA with the best recall performance. Confidence interval of the overall estimated effect does not overlap with 0.

As for the eM1 model, the M5 meta-analytic model estimated the overall effect-size of a difference in metacognitive sensitivity between YA and OA for eFOK. It showed lower gamma correlations in OA compared to YA, *g* = 0.78 [0.56, 1.00], *p* < 0.001 (see Fig. 6A). The total heterogeneity analysis revealed a non-significant Q-statistic (*Q*(df = 5) = 4.32, *p* = 0.504). The M6 model estimated the overall effect-size of a difference in recall between YA and OA for eFOK. It showed an overall large effect size of *g* = 1.33 [1.09, 1.56], *p* < 0.001 (see Fig. 6B) resulting in a higher performance in recall for YA compared to OA.

Models M7a and M7b were performed on half of the sample size. For each study both YA and OA were split into two groups according to their recall performance (median split). M7a compared eFOK for OA with the highest recall performance and YA with the lowest recall performance. On the contrary M7b compared eFOK for OA with the lowest recall performance and YA with the highest recall performance. Both M7a and M7b revealed a significant effect. Although the estimated effect size was lower for M7a, *g* = 0.52 [0.14, 0.91], *p* < 0.001 than for M7b, *g* = 1.06 [0.70, 1.42], *p* < 0.001 (see Fig. 4C,D), confidence intervals overlap. Note that these intervals are large as each model only includes half of the participants.

**Figure 7:**
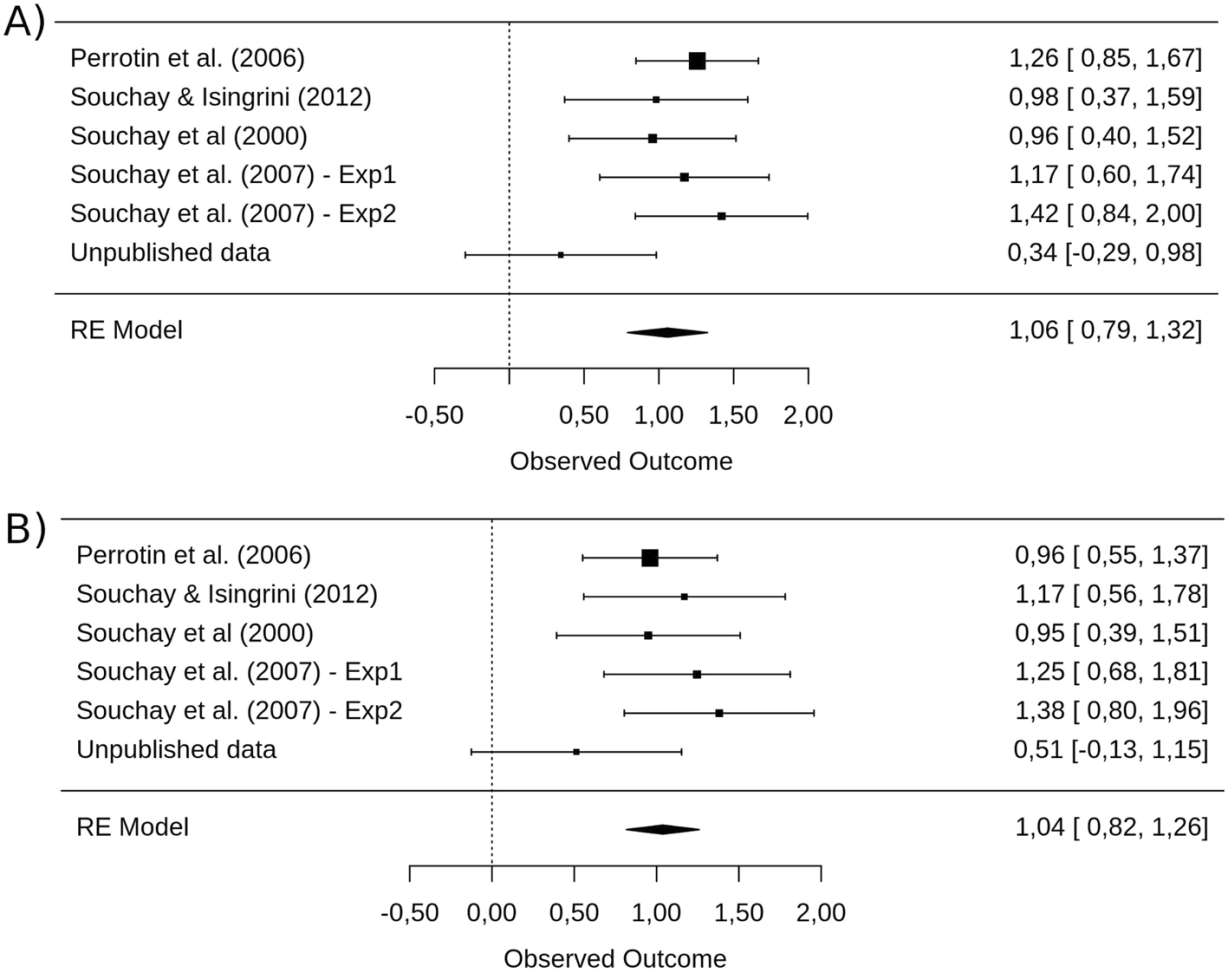
**(A)** Forrest plot of the effect of eFOK deficit in OA using the Hamann coefficient. Confidence interval of the overall estimated effect does not overlap with 0. **(B)** Forrest plot of the effect of eFOK deficit in OA using type-II d’. Confidence interval of the overall estimated effect does not overlap with 0.

As exploratory analyses, we also conducted two other meta-analyses similar to M5. Instead of using corrected-gamma as a measure of metacognitive sensitivity, we used the Hamann coefficient and type-II d’. Type-II d’ was adjusted for extreme hits and false alarms by replacing rates of 0 with 0.5/n and rates of 1 with (n − 0.5)/n where n is the number of signal or noise trials^45^. As for the M5 model, the model with Hamann coefficient showed an overall effect of *g* = 1.06 [0.79, 1.32], *p* < 0.001 with no significant heterogeneity *Q*(df = 5) = 7.52, *p* = 0.185, as well as the model with type-II d’, *g* = 1.04 [0.82, 1.26], *p* < 0.001, *Q*(df = 5) = 4.88, *p* = 0.431 (see Fig. 7A,B).

##### Non-preregistered analyses

As the two meta-analyses splitting participants according to recall performance led to unclear results due to the small overlap between confidence intervals, we conducted a complementary analysis. The global meta-analysis revealed a moderator effect of recognition performance (and a trend for recall) going toward the idea that memory function is involved in eFOK sensitivity. This moderation by recognition however cannot disentangle between a proper memory-metamemory interaction, a spurious relationship due to gamma, or more likely both factors (because of the trend effect of recall). To see whether recognition performance better explains eFOK than recall also in our aggregated dataset, we performed the same split-analysis using recognition performance. The two datasets were therefore created according to the median split.

**Figure 8:**
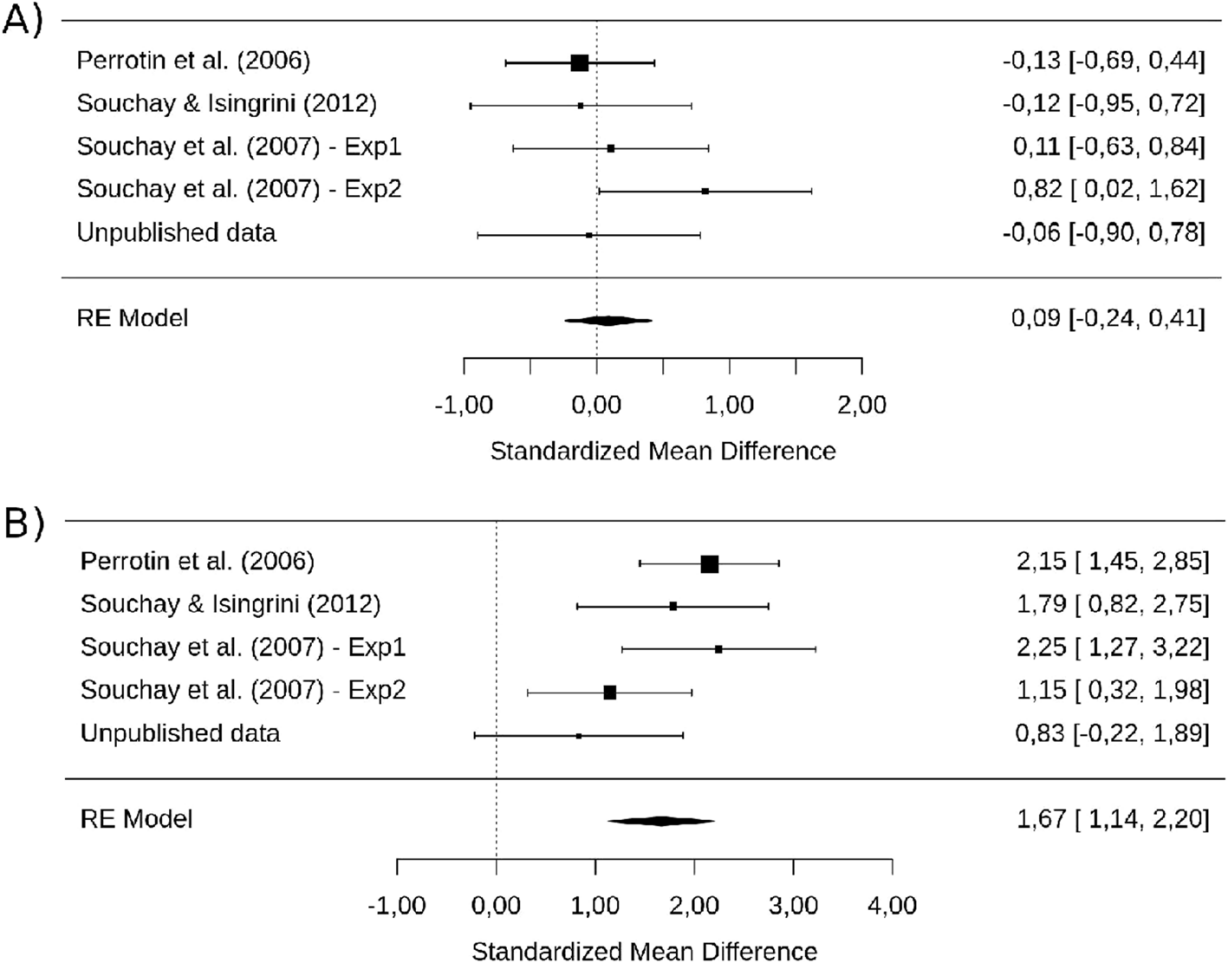
**(A)** Forrest plot of the effect of eFOK deficit in OA for half of OA with the best recognition performance and half of YA with the worst recognition performance. Confidence interval of the overall estimated effect overlaps with 0. (**B**) Forrest plot of the effect of eFOK deficit in OA for half of OA with the worst recognition performance and half of YA with the best recognition performance. The confidence interval of the overall estimated effect does not overlap with 0.

Akin to M7a and M7b, we created M8a and M8b. M8a showed a non-significant overall estimated effect, *g* = 0.09 [−0.24, 0.41], *p* = 0.601 whereas the effect for M8b was significant, *g* = 1.67 [1.14, 2.20], *p* < 0.001 (see Fig. 8A, B). Moreover, as confidence intervals do not overlap with each other, both models estimated different overall effects significantly.

## Discussion

We conducted a systematic review and a meta-analysis of eFOK and sFOK in aging. We confirmed our main hypothesis of a preserved sFOK sensitivity and a moderately impaired eFOK sensitivity in aging (*g* = 0.53). The large heterogeneity observed in the qualitative and the quantitative analyses is the result of a variety of methodologies in the different studies that were mostly used to control for memory differences between OA and YA. Such variability is not present in sFOK studies and OA have the same (or sometimes even better) performance in semantic memory tasks as YA. We also found that the aggregated dataset meta-analysis slightly inflated the estimation of the eFOK deficit in aging (from *g* = 0.53 to *g* = 0.78), probably due to the fact there was no attempt to control for first order performance (also confirmed by the non-significant heterogeneity for this meta-analytic model).

We investigated whether episodic memory performance explains the eFOK deficit observed in OA. Moderator analyses revealed that recall (as a trend) and especially recognition reduced the overall effect size. The analysis of the model’s intercept suggests that when there is no difference between OA and YA in terms of recognition performance, the eFOK difference vanishes. Using aggregated dataset, reducing or increasing group difference in recall memory by selecting best/worst 50% of OA and worst/best 50% YA respectively only slightly reduced (from *g* = 0.78 to *g* = 0.52; see Fig. 4C) or enlarged (from *g* = 0.78 to *g* = 1.06 ; see Fig. 4D) the age-effects and the two models did not differ significantly. These analyses included only half of the sample and reduced statistical power could explain the overlap in confidence intervals of the effect sizes. Nonetheless, when performing the same median split analyses using recognition performance, modulations of the overall effect size were higher (from *g* = 0.78 to *g* = 0.09, see Fig. 8A; and from *g* = 0.78 to *g* = 1.67, see Fig. 8B). The model that decreased the recognition gap between groups led to non-significant results. As such, when memory performance is equal across groups, the eFOK deficit in OA does not exist anymore.

Differences in recall and recognition therefore account for the different effects on eFOK sensitivity. This could be due to two reasons. First, we tackle contamination between measures of first order and second order performance, that is the age difference in accuracy could be a statistical artifact. Secondly, we discuss the possibility that there is a psychologically real reason why diminished memory performance leads to impoverished eFOK accuracy.

First, FOK sensitivity is calculated from recognition performance which explains that the larger the metacognitive sensitivity measure is biased by first order performance, the larger the estimated deficit. This is in line with our exploratory analysis showing a larger estimated effect using type-II d’ than gamma correlation. Theoretically, type-II d’ is less influenced by bias, as signal detection theory is proposed to distinguish between bias and sensitivity (but see^46^). However, first-order performance is more likely to influence this measure as type-II d’ assumes that the distributions for “correct” and “incorrect” are Gaussian with equal variance which is rarely the case at the type-II level^47^.

Metacognitive sensitivity measures have also been shown to be influenced by guessing during the recognition task in modeling on hypothetical data^48^ . Gamma was drastically reduced as a function of guessing (i.e., for lower levels of knowledge) even when the relationship between first and second order performance was held constant in the model. The memory deficit in aging is variable but overall, we suggest that OA conform to this notion of middle-to-low performance as described by Vuorre and Metcalfe^48^.

Secondly, we propose that memory function is intrinsically linked to the capacity to make accurate metamemory judgements. If the eFOK sensitivity difference in aging is due to an interaction between a metacognitive sensitivity measure and an episodic memory deficit, a pure memory hypothesis is likely also at play. FOKs are performed after recall. Thus, these judgments are undoubtedly directly influenced by the recall process (e.g., partial information available at this stage,^34,49,50^. For example, Eakin & Hertzog^24^ showed that FOKs were more strongly correlated with recall than with recognition responses, both in YA and in OA. Moreover, in both eFOK and sFOK tasks, Mazancieux et al.^51^ showed that metacognitive efficiency (a measure of metacognitive sensitivity that controls for recognition) variability is more strongly correlated with recall than recognition. OA are proposed to have a trade-off between a deficit in the recollection process (also at play in recall tasks) and a preserved familiarity process^52,53^. If the output of the retrieval process during recall leads to no information on which to base FOKs, OA are not able to anticipate recognition.

Importantly, we found that the eFOK deficit in aging is mainly explained by lower recognition in OA, and not recall. This points more in the direction of a contamination between first and second order performance than a genuine problem of memory function, but this is something which needs further consideration. Therefore, we argue here for new studies that better control recognition performance between YA and OA. In the literature on confidence judgments, two main methods are used to achieve such control. The first is to use a metacognitive sensitivity measure that takes into account first-order performance such as the meta-d’/d’ ratio^54–56^ also known as metacognitive efficiency even though small dependencies between d’ and the meta-d’/d’ ratio also exists particularly for low first order performance^57^. The second method involves the experimental protocol that is used. Staircase procedures are often used to equate first order performance across groups or tasks although this has also been shown to inflate estimations of metacognitive efficiency^58^. Nonetheless, we propose that extensions of such protocols could be used in the FOK literature by for instance changing the distractors for trial n + 1 according to performance at trial n, or by manipulating other variables online which are critical for first order performance, such as study time, and retention interval.

Another simple solution would also be to measure episodic memory performance on a separate, perhaps standardized task, such that the episodic memory function and the FOK measure are not taken from the same task, thus sidestepping some of the issues of contamination between the measures statistically. Interestingly, if the eFOK was merely a statistical artifact or measurement issue, it would be expected that where OA semantic memory performance fell below that of YA people to a similar magnitude as episodic memory, we should also see parallels in sFOK performance: sFOK sensitivity should likewise be impaired where first-order performance is deficient.

Finally, the eFOK meta-analysis funnel plot was asymmetric suggesting a publication bias. This is not surprising if the eFOK deficit in aging is mainly explained by a contamination between first (here the recognition task) and second order performance, considering that OA are mainly impaired on recollection and can still solve recognition tasks based on familiarity^53^. According to task difficulty (e.g., number and type of distractors in the recognition task), substantial between-experiment variability can occur.

As a final note, we would like to point out that this review focused on FOK sensitivity in advanced age but not on the gradual changes of metacognitive sensitivity with aging which could lead to complementary results. For example, previous work included a middle-age group (from 40 to 52 years old^9^ on sFOK), compared groups from all age-ranges (from 18 to 83^14^ on retrospective judgements), or investigated the correlation between metacognitive efficiency of retrospective judgements and age^59^.

In sum, our meta-analysis points to age-related differences in eFOK sensitivity. This deficit is clear both when comparing YA and OA eFOK performance, but also when comparing sFOK and eFOK performance within the OA. It seems to us that this deficit is due to (1) confounds related to the interaction between the gamma calculation, guessing, and lower memory performance, (2) lower memory performance due to a specific recollection deficit arising at the recall stage which affects the information on which to base metamemory evaluations. The key factor at play in these two hypotheses relates to first order performance, which also explains the pattern of preserved sFOK found in this article (where there was no such group difference). We believe that future studies should take into account this first order performance variability across groups in order to investigate potential parts of metacognition that could differ across YA and OA.

## Methods

### Systematic review and global meta-analysis

#### Selection and inclusion

The systematic review and meta-analysis were conducted following the PRISMA guidelines and recommendations^60^. Summary of the selection steps are described in Fig. 2.

#### Identification

Records published as of October 15th 2020 were identified from PubMed and Web of Sciences online databases. Two searches were carried out each using the keyword “feeling-of-knowing” associated first with “older adults” and then with “aging”. Additional reports were identified by checking references in selected papers. No time limit was set regarding the year of publication. After records were identified, duplicates were removed.

#### Screening

Articles were first selected on the basis of their title and abstract. Records not dealing with aging or not using the FOK procedure were excluded. We also withdrew records which were not published papers or not original research reports.

#### Eligibility criteria

For inclusion in the systematic review, full texts of selected articles were inspected. Only records which met eligibility criteria were included. To be eligible, records had to be original research articles written in French or in English. Inclusion criteria also comprised comparison of a group of YA and a group of OA using a sFOK or eFOK standard paradigm. In an eFOK paradigm, participants predict the likelihood of future recognition of newly learnt material (e.g., pictures or words). We excluded studies in which the metacognitive judgment was called a ‘feeling of knowing’ but did not consist in a prediction of future recognition performance (e.g.,^61^; Experiments 2 and 3 of^42^ or was not a standard FOK procedure^62^). To be eligible, studies also had to describe performance using a measure of metacognitive sensitivity (e.g., gamma correlation, Hamman correlation, type-2 d’). In order to perform statistical analyses, an additional eligibility criterion for inclusion in the meta-analysis was the description of sample sizes, means and standard deviations for the metacognitive sensitivity measure.

As introduced briefly above and discussed in detail below, several studies included multiple comparison groups with the aim of equating first order performance between YA and OA. Sacher et al.^39^ includes three YA groups and one OA group. Because all three YA groups are of interest for our main memory hypothesis on eFOK, we decided to include them all in the meta-analysis. To consider the fact that these 3 comparisons include the same OA group, we used a hierarchical meta-analytic model that takes into account the ‘effect’ variability within a particular study (see “Statistical analyses” section for more details). Hertzog, Dunlosky & Sinclair^26^ compared two groups of OA tested either after a 48-h or 30-min delay with one group of YA tested after a seven-day delay. However, as data for the 30-min condition was not available in the article, we decided to exclude this condition from the current meta-analysis.

Several studies also included other within-subject manipulations but we did not focus on these manipulations. In such studies^40^, we included the overall performance instead of data for each specific condition. Thomas et al. also added between-subject manipulations. In their Experiment 2, participants attempted recall of partial information either before or after the FOK. In Experiment 3, participants had a time limit to either perform their FOK or to retrieve partial information. For each experiment, data for both conditions were included separately in the analysis. In Eakin & Hertzog^24^, overall performance was not provided. Data for each condition was used and the comparison between the two groups was made for each condition. Finally, Eakin & Hertzog^24^ proposed two procedures (intralist and extralist cueing conditions). As this extralist condition moves away from the classical FOK paradigm, we decided to not include this data in the meta-analysis.

#### Statistical analyses

Analyses were performed on the measure of metacognitive sensitivity using four models (M1 to M4). The FOK scales were different across studies (see Table 1), however all studies used gamma correlations as a measure of sensitivity that is not biased by the type of judgements scale^63^. Hedges’s G was calculated to measure the effect size of age on metacognitive sensitivity. All analyses were performed using R software and multilevel meta-analysis models were carried out with the *metafor* package.

Our first meta-analytic model (M1) estimated the overall effect-size of a difference in metacognitive sensitivity between YA and OA taking also variability into account at three levels: the study level, the experiment level, and the effect level. We ran two versions of this model: one for eFOK (eM1) and one for sFOK (sM1).

As preregistered, we tested the hypothesis that eFOK effect can be reduced by controlling for memory. Two models were created: one testing recognition performance as a moderator and the other testing recall performance as a moderator, as they both capture similar but also different aspects of the confounds. Controlling for recognition performance allows an estimation of OA’s eFOK deficit that could result from both (1) the intrinsic relationship between episodic memory and metamemory (i.e., metamemory sensitivity is based on memory processes such as recollection) and (2) confounds in statistical/mathematical quantification of metacognitive sensitivity (i.e., the use of gamma as it directly takes into account recognition performance in its calculation). On the other hand, as FOKs are performed after a recall attempt, controlling for recall would control for the intrinsic relationship between episodic memory and metamemory. That is, FOKs are influenced by the ability to recall the target or specific information about it. As an example, Mazancieux et al.^52^ found a correlation between recall and metacognitive efficiency in both eFOK and sFOK. In short, whereas recognition performance may contribute to a statistical artifact in measures of metacognitive sensitivity as well as looking at genuine mnemonic factors, the recall measure captures something slightly different, since it does not contribute directly to the measure of metacognitive sensitivity, and captures memory function at the point of making the FOK judgment, not subsequent to it.

For the M2 model, we calculated Hedge’s g effect sizes for the difference between YA and OA in recall (using means and standard deviations). This model was identical to M1e but included recall effect sizes as a moderator. Because of missing information in several articles, only 16 out of 22 effects (630 YA and 722 OA) were included in this analysis. For the M3 model, the same procedure was used with recognition performance including 14 out of 22 effects (536 YA and 606 OA). Finally, our last preregistered moderator to be tested was the type of recognition (e.g., 2AFC, yes/no). For the M4 model, we added to the M1e model a nominal moderator corresponding to the type of recognition task used for each effect (e.g., two al.

All moderator analyses (M2, M3, M4) were performed in case of significant heterogeneity in M1e. This assessment was carried out using the Q-sta­tistic^64^ and the I^2^ index, which corresponds to the percentage of the total variation due to between-studies variability^65^. I^2^ values above 50% are considered a large amount of heterogeneity.

Publication bias was assessed using funnel plots (one for eFOK and one for sFOK) of observed outcomes according to corresponding standard errors^66^. The plot asymmetry was tested using an adapted Egger’s test for multilevel models.

### Analyses of individual scores from aggregated dataset

#### Dataset description

Data was taken from five published (^28,31^; two studies from^19,27^ and 1 unpublished studies conducted in our lab (note that the 5 published studies were also included in the global meta-analysis). The unpublished data included 20 OA and 18 YA in a list recognition task. In total, data included recall performance and counts of recognized and unrecognized items given a yes or no FOK, producing a 2 × 2 table. This structure allowed us to calculate gamma correlation (for our preregistered confirmatory hypotheses) but also Hamman coefficient and type-II d’ (according to our preregistered exploratory hypotheses). Because several participants had no data in at least one of the boxes of 2 × 2 of the table, we calculated the corrected gamma, Hamman, and type-II d’. Note that we found some discrepancies between calculated means and means reported in corresponding articles as a probable consequence of disparities in methods used for correction of scores or rounding methods.

This aggregated dataset of individual data allows us to have more sensitive tests of our hypothesis. Therefore, we aimed to (1) reproduce findings of the global meta-analysis, (2) compare gamma across 50% of the OA with the best recall performance and 50% of the YA with the worst recall performance, (3) compare gamma for OA and YA with recall performance as a covariable, and (4) explore the effect of type of metacognitive sensitivity measure. For our confirmatory hypothesis (points 2 and 3), we expect to find a lower eFOK difference between YA and OA.

#### Statistical analyses

Analyses were also performed using the R software with the *metafor* package. Hedges’s G was calculated to measure the effect size of age on metacognitive sensitivity for each study. Several meta-analytic models were performed. The first (M5) estimated the overall effect-size of a difference in eFOK metacognitive sensitivity between YA and OA as a reproduction of the global meta-analysis. Model M6 estimated the overall effect-size of a difference in recall performance between YA and OA. Then, the following models were run in order to test the influence of memory performance in metacognitive sensitivity. Models M7a and M7b were performed on half of the sample size. For each study both YA and OA were split into 2 groups according to their recall performance (median split). M7a compared eFOK for OA with the highest recall performance and YA with the lowest recall performance. On the contrary M7b compared eFOK for OA with the lowest recall performance and YA with the highest recall performance. As preregistered, we suppose a reduction of the overall estimated effect in M7a compared to M7b.

## Data Availability

The method and analyses were pre-registered on the Open Science Framework (https://osf.io/5hkpt/). Raw data extracted from each selected article and analysis scripts are available on GitHub (https://github.com/amazancieux/eFOKaging_review).
